# DA-TransUNet: integrating spatial and channel dual attention with transformer U-net for medical image segmentation

**DOI:** 10.3389/fbioe.2024.1398237

**Published:** 2024-05-16

**Authors:** Guanqun Sun, Yizhi Pan, Weikun Kong, Zichang Xu, Jianhua Ma, Teeradaj Racharak, Le-Minh Nguyen, Junyi Xin

**Affiliations:** ^1^ School of Information Engineering, Hangzhou Medical College, Hangzhou, China; ^2^ School of Information Science, Japan Advanced Institute of Science and Technology, Nomi, Japan; ^3^ Department of Electronic Engineering, Tsinghua University, Beijing, China; ^4^ Department of Systems Immunology, Immunology Frontier Research Institute (IFReC), Osaka University, Suita, Japan; ^5^ Faculty of Computer and Information Sciences, Hosei University, Tokyo, Japan; ^6^ Zhejiang Engineering Research Center for Brain Cognition and Brain Diseases Digital Medical Instruments, Hangzhou Medical College, Hangzhou, China; ^7^ Academy for Advanced Interdisciplinary Studies of Future Health, Hangzhou Medical College, Hangzhou, China

**Keywords:** U-net, medical image segmentation, dual attention, transformer, deep learning

## Abstract

Accurate medical image segmentation is critical for disease quantification and treatment evaluation. While traditional U-Net architectures and their transformer-integrated variants excel in automated segmentation tasks. Existing models also struggle with parameter efficiency and computational complexity, often due to the extensive use of Transformers. However, they lack the ability to harness the image’s intrinsic position and channel features. Research employing Dual Attention mechanisms of position and channel have not been specifically optimized for the high-detail demands of medical images. To address these issues, this study proposes a novel deep medical image segmentation framework, called DA-TransUNet, aiming to integrate the Transformer and dual attention block (DA-Block) into the traditional U-shaped architecture. Also, DA-TransUNet tailored for the high-detail requirements of medical images, optimizes the intermittent channels of Dual Attention (DA) and employs DA in each skip-connection to effectively filter out irrelevant information. This integration significantly enhances the model’s capability to extract features, thereby improving the performance of medical image segmentation. DA-TransUNet is validated in medical image segmentation tasks, consistently outperforming state-of-the-art techniques across 5 datasets. In summary, DA-TransUNet has made significant strides in medical image segmentation, offering new insights into existing techniques. It strengthens model performance from the perspective of image features, thereby advancing the development of high-precision automated medical image diagnosis. The codes and parameters of our model will be publicly available at https://github.com/SUN-1024/DA-TransUnet.

## 1 Introduction

Machine learning and deep learning techniques have emerged as powerful tools in biomedical research, revolutionizing disease diagnosis, treatment planning, and personalized medicine (Le, 2024; Tran and Le, 2024). Medical image segmentation is the process of delineating regions of interest within medical images for diagnosis and treatment planning. It serves as a cornerstone in medical image analysis. Manual segmentation is both accurate and affordable for pathology diagnosis but vital in standardized clinical settings. Conversely, automated segmentation ensures a reliable and consistent process, boosting efficiency, cutting down on labor and costs, and preserving accuracy. Consequently, there is a substantial demand for exceptionally accurate automated medical image segmentation technology within the realm of clinical diagnostics. However, medical image segmentation faces unique challenges, such as the need for precise delineation of complex anatomical structures, variability across patients, and the presence of noise and artifacts in the images (Tran et al., 2023). These challenges necessitate the development of advanced segmentation techniques that can capture fine-grained details while maintaining robustness and efficiency.

In the last decade, the traditional U-net structure has been widely employed in numerous segmentation tasks, yielding commendable outcomes. Notably, the U-Net model (Ronneberger et al., 2015), along with its various enhanced iterations, has achieved substantial success. ResUnet (Diakogiannis et al., 2020) emerged during this period, influenced by the residual concept. Similarly, UNet++ (Zhou et al., 2018) emphasizes enhancements in skip connections. Moving beyond these CNN-based approaches, the Transformer architecture introduces a completely new perspective. The transformer (Vaswani et al., 2017), originally developed for sequence-to-sequence modeling in Natural Language Processing (NLP), has also found utility in the field of Computer Vision (CV). ViTs segment images into patches and input their embeddings into a transformer network for strong performance. (Dosovitskiy et al., 2020). This signifies a trend of shifting from traditional CNN models to more flexible Transformer models. While the above-mentioned U-Net structures have enhanced the capabilities of models in segmentation tasks (Ronneberger et al., 2015; Zhou et al., 2018; Diakogiannis et al., 2020), they do not integrate the more powerful feature extraction abilities inherent in the Transformer and attention mechanisms, which limits their potential for further improvement. On the one hand, several studies have made progress in image segmentation by leveraging Dual Attention (DA) mechanisms for both channels and positions. The Dual Attention Network (DANet) utilizes a Position Attention Block (PAM) and Channel Attention Block (CAM) from the DA Network for natural scene image segmentation (Fu et al., 2019). This research primarily focuses on scene segmentation and does not explore the unique characteristics of medical imagery. Also, DAResUnet (Shi et al., 2020) introduces a dual attention block combined with a residual block (Res-Block) in a U-net architecture for medical image segmentation, demonstrating significant improvements in this domain. However, in the realm of medical image segmentation, existing models, including those employing Dual Attention mechanisms, have not yet extensively explored the optimal integration of Dual Attention with Transformer models for enhanced feature extraction; this oversight represents a significant research opportunity in the task of medical image segmentation. Therefore, addressing this gap and optimizing the integration of Transformers and Dual Attention mechanisms in the context of medical image segmentation poses a significant challenge for future research in the field.

To overcome the above drawbacks, recent studies have explored the application of Transformer models in medical image segmentation. Inspired by ViTs, TransUNet (Chen et al., 2021) further combines the functionality of ViTs with the advantages of U-net in the field of medical image segmentation. Specifically, it employs a transformer’s encoder to process the image and employs CNN and hopping connections for accurate up-sampling feature recovery, yet it neglects image-specific features like position and channel. These aspects are crucial for capturing the nuanced variations and complex structures often present in medical images, which are essential for accurate diagnosis and analysis. Swin-Unet (Cao et al., 2022) combines the Swin-transform block with the U-net structure and achieves good results. Yet, adding extensive Transformer blocks inflates the parameter count without significantly improving results. This study merely stacked multiple Transformers to enhance models, resulting in inflated parameters and computational complexity with marginal gains in performance. Moreover, some studies have specifically focused on incorporating position and channel attention mechanisms in medical image segmentation. For instance, DA-DSUnet has been applied to head-and-neck tumor segmentation, but it doesn’t combine Position Attention Module (PAM) and Channel Attention Module (CAM), nor does it discuss the potential filtering role of DA blocks in skip connections (Tang et al., 2021). Additionally, it doesn’t leverage ViT for feature extraction. Another example is research on brain tumor segmentation, which, while applying DA blocks, limits its scope to brain tumors without validating other types of medical images (Sahayam et al., 2022). These studies integrate DA blocks with other blocks but do not thoroughly explore the role of DA in skip connections or optimize DA blocks for the unique intricacies of medical imaging.

However, Despite the progress made by these transformer-based approaches, they often overlook the importance of integrating image-specific features, such as position and channel information, which are crucial for capturing the nuanced variations and complex structures in medical images. Moreover, the existing methods that incorporate dual attention mechanisms have not been optimized for the unique characteristics of medical imagery, leaving room for further improvement. To address these limitations, we propose DA-TransUNet, which strategically integrates the Dual Attention Block (DA-Block) into the transformer-based U-Net architecture, specifically tailored for medical image segmentation.

In this research, our proposed model DA-TransUNet is an innovative approach for medical image segmentation that integrates the Transformer mechanism, specifically the Vision Transformer (ViT) and a Dual Attention (DA) mechanism within a U-Net architecture. First, the Transformer ViT is combined with DA in the encoder of the U-Net structure, enhancing feature extraction capabilities by leveraging the detailed characteristics of medical images. This integration allows the model to capture both local and global contextual information, which is essential for accurate segmentation of complex anatomical structures. Then, to further refine feature extraction tailored to medical images, DA is optimized for specific channels and incorporated into every module of the skip connections, enabling the model to effectively filter out irrelevant information and focus on the most discriminative features. The skip connections pass the shallow positional information from the encoder, while the DA module refines the crucial detailed features. This targeted optimization is substantiated by extensive ablation studies, demonstrating its significance in improving the model’s performance. Lastly, this architecture has been rigorously tested across five medical image segmentation datasets and extensive ablation studies, demonstrating its effectiveness and superiority (Candemir et al., 2013; Jaeger et al., 2013; Bernal et al., 2015; Landman et al., 2015; Tschandl et al., 2018; Codella et al., 2019; Jha et al., 2020; Jha et al., 2021).

The main contributions of this article are summarized as follows:

1) The model of DA-TransUnet is proposed by integrating Transformer ViT and Dual Attention in U-net architecture’s encoder and skip connections. This design enhances feature extraction capabilities in better extracting detailed features of medical images.
2) We propose an optimized Dual Attention (DA) Block that is designed for medical image segmentation with two key enhancements: the optimization of intermediate channel configurations within the DA block, and its integration into each skip-connection layer for effectively filtering irrelevant information. These are validated through comprehensive ablation experiments.
3) The segmentation performance and generalization ability of DA-TransUnet are validated on five medical datasets. In comparison to recent related studies, DA-TransUnet exhibits superior results in medical image segmentation, demonstrating its effectiveness in this field.

The rest of this article is organized as follows. Section 2 reviews the related works of automatic medical image segmentation, and the description of our proposed DA-TransUNet is given in Section 3. Next, the comprehensive experiments and visualization analyses are conducted in Section 4. Finally, Section 5 makes a conclusion of the whole work.

## 2 Related work

### 2.1 U-net model

Recently, attention mechanisms have gained popularity in U-net architectures (Ronneberger et al., 2015). For example, Attention U-net incorporates attention mechanisms to enhance pancreas localization and segmentation performance (Oktay et al., 2018); DAResUnet integrates both double attention and residual mechanisms into U-net (Shi et al., 2020); Attention Res-UNet explores the substitution of hard-attention with soft-attention (Maji et al., 2022); Sa-unet incorporates a spatial attention mechanism in U-net (Guo et al., 2021). Following this, TransUNet innovatively combines Transformer and U-net structure (Chen et al., 2021). Building on TransUNet, TransU-Net++ incorporates attention mechanisms into both skip connections and feature extraction (Jamali et al., 2023). Swin-Unet (Cao et al., 2022) improves by replacing every convolution block in U-net with Swin-Transformer (Liu et al., 2021). DS-TransUNet proposes to incorporate the tif module (which is a multi-scale module using Transformer) to the skip connection to improve the model (Lin et al., 2022). AA-transunet leverages Block Attention Model (CBAM) and Deep Separable Convolution to further optimize TransUNet (Yang and Mehrkanoon, 2022). TransFuse uses dual attention Bifusion blocks and AG to fuse features of two different parts of CNN and Transformer (Zhang et al., 2021). Numerous attention mechanisms have been added to U-net and TransUNet models, yet further exploration is warranted. Diverging from prior approaches, our experiment introduces a dual attention mechanism and Transformer module into the traditional U-shaped encoder-decoder and skip connections, yielding promising results.

### 2.2 Application of skip connections in medical image segmentation modeling

Skip connections in U-net aim to bridge the semantic gap between the encoder and decoder, effectively recovering fine-grained object details (Drozdzal et al., 2016; He et al., 2016; Huang et al., 2017). There are three primary modifications to skip connections: firstly, increasing their complexity (Azad et al., 2022a). U-Net++ redesigned the skip connection to include a Dense-like structure in the skip connection (Zhou et al., 2018), and U-Net3++(Huang et al., 2020) changed the skip connection to a full-scale skip connection. Secondly, RA-UNet introduces a 3D hybrid residual attention-aware method for precise feature extraction in skipped connections (Jin et al., 2020). The third is a combination of encoder and decoder feature maps: An alternative extension to the classical skip connection was introduced in BCDU-Net with a bidirectional convolutional long-term-short-term memory (LSTM) module was added to the skip connection (Azad et al., 2019). Aligning with the second approach, we integrate Dual Attention Blocks into each skip connection layer, enhancing decoder feature extraction and thereby improving image segmentation accuracy.

### 2.3 The use of attentional mechanisms in medical images

Attention mechanisms are essential for directing model focus towards relevant features, thereby enhancing performance. In recent years, dual attention mechanisms have seen diverse applications across multiple fields. In scene segmentation, the Dual Attention Network (DANet) employs position and channel attention mechanisms to improve performance (Fu et al., 2019). A modularized DANs framework is presented that adeptly merges visual and textual attention mechanisms (Nam et al., 2017). This cohesive approach enables selective focus on pivotal features in both types of data, thereby improving task-specific performance. Additionally, the introduction of the Dual Attention Module (DuATM) has been groundbreaking in the field of audio-visual event localization. This model excels at learning context-aware feature sequences and performing attention sequence comparisons in tandem, effectively incorporating auditory-oriented visual attention mechanisms (Si et al., 2018). Moreover, dual attention mechanisms have been applied to medical segmentation, yielding promising results (Shi et al., 2020). The Multilevel Dual Attention U-net for Polyp Segment combines dual attention and U-net in medical image segmentation (Cai et al., 2022). While significant progress has been made in medical image segmentation, there is still ample room for further research to explore the potential of position and channel attention mechanism in the field of medical image segmentation.

## 3 Methods

**FIGURE 1:**
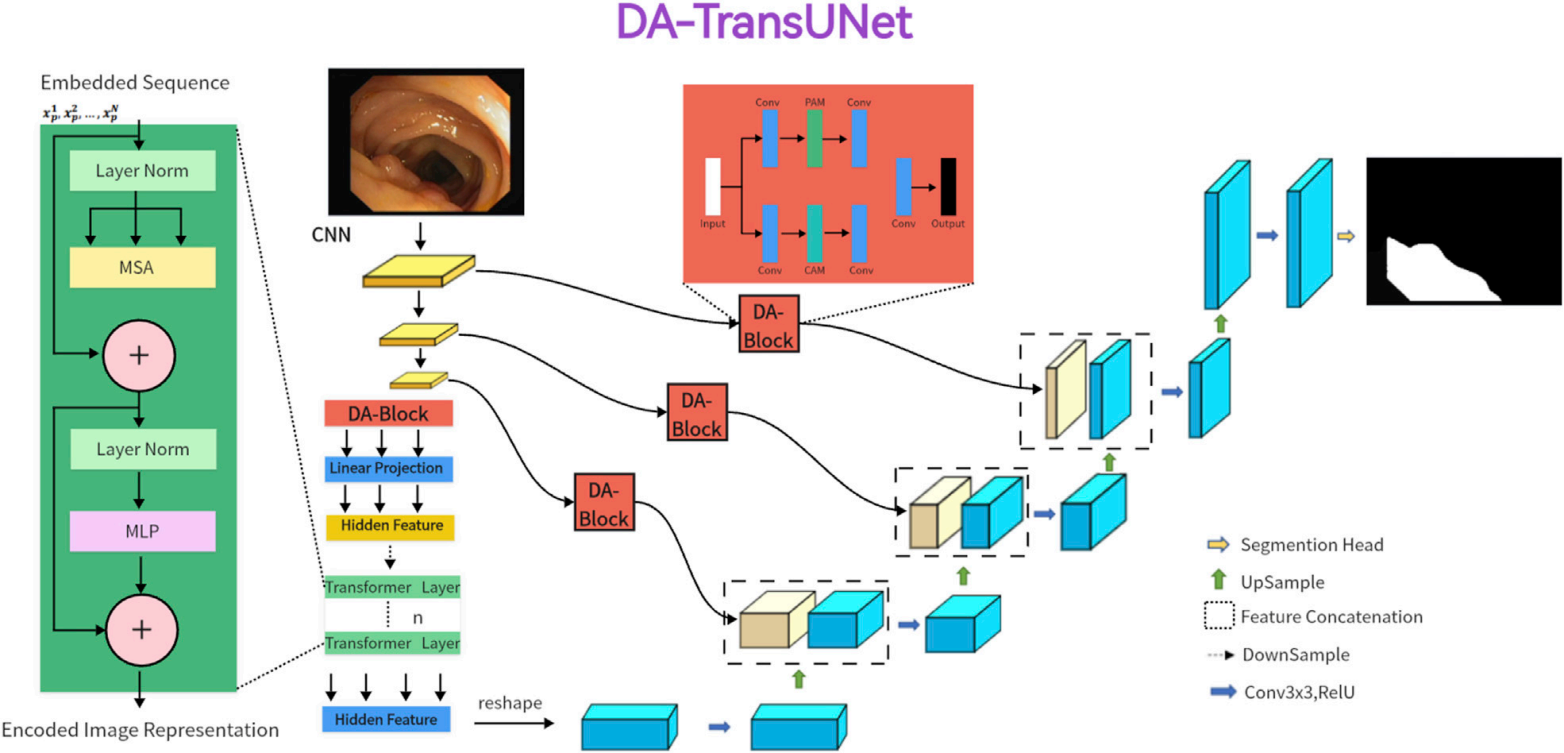
Illustration of the proposed dual attention transformer U-Net(DA-TransUNet). For the input medical images, we feed them into an encoder with transformer and Dual Attention Block (DA-Block). Then, the features of each of the three different scales are purified by DA-Block. Finally, the purified skip connections are fused with the decoder, which subsequently undergoes CNN-based up-sampling to restore the channel to the same resolution as the input image. In this way, the final image prediction result is obtained.

In the subsequent section, we propose the DA-TransUNet architecture, illustrated in Figure 1. We start with a comprehensive overview of the architecture. Next, we detailed the architecture’s key components in the following order: the dual attention blocks (DA-Block), the encoder, the skip connections, and the decoder.

### 3.1 Overview of DA-TransUNet

In Figure 1, the architecture of DA-TransUNet is presented. The model comprises three core components: the encoder, the decoder, and the skip connections. In particular, the encoder fuses a conventional convolutional neural network (CNN) with a Transformer layer and is further enriched by the DA-Block, which are exclusively introduced in this model architecture. In contrast, the decoder primarily employs conventional convolutional mechanisms. For the optimization of skip connections, DA-Blocks serve as pivotal components within the DA-TransUNet architecture. DA-Blocks filter irrelevant information in skip connections, enhancing image reconstruction accuracy. In summary, in contrast to traditional convolutional approaches and the extensive use of Transformers, DA-TransUNet uniquely leverages DA-Blocks for the extraction and utilization of image-specific features of position and channel. This strategic incorporation significantly elevates the overall performance of the model.

Compared to traditional U-Net architectures, DA-TransUNet integrates the Transformer layer in the encoder to capture global dependencies, while the U-Net relies solely on convolutional layers for local feature extraction. Moreover, the inclusion of DA-Blocks in the encoder and skip connections sets DA-TransUNet apart from both U-Net and Transformer-based models. These DA-Blocks enable the extraction and utilization of image-specific position and channel features, enhancing the model’s ability to capture fine-grained details crucial for medical image segmentation.

To elucidate the rationale behind our proposed DA-TransUNet model’s design, it’s imperative to consider the limitations and strengths of both U-Net architectures and Transformers in the context of feature extraction. While Transformers excel in global feature extraction through their self-attention mechanisms, they are inherently limited to unidirectional focus on positional attributes, thus neglecting multi-faceted feature perspectives. On the other hand, traditional U-Net architectures are proficient in local feature extraction but lack the capability for comprehensive global contextualization. To address these constraints, we integrate DA-Blocks both preceding the Transformer layers and within the encoder-decoder skip connections. This achieves two goals: firstly, it refines the feature map input to the Transformer, enabling more nuanced and precise global feature extraction; secondly, the DA-Block in the skip connections optimize the transmitted features from the encoder, facilitating the decoder in reconstructing a more accurate feature map. Thus, our proposed architecture amalgamates the strengths and mitigates the weaknesses of both foundational technologies, resulting in a robust system capable of image-specific feature extraction.

### 3.2 Dual attention block (DA-Block)

**FIGURE 2:**
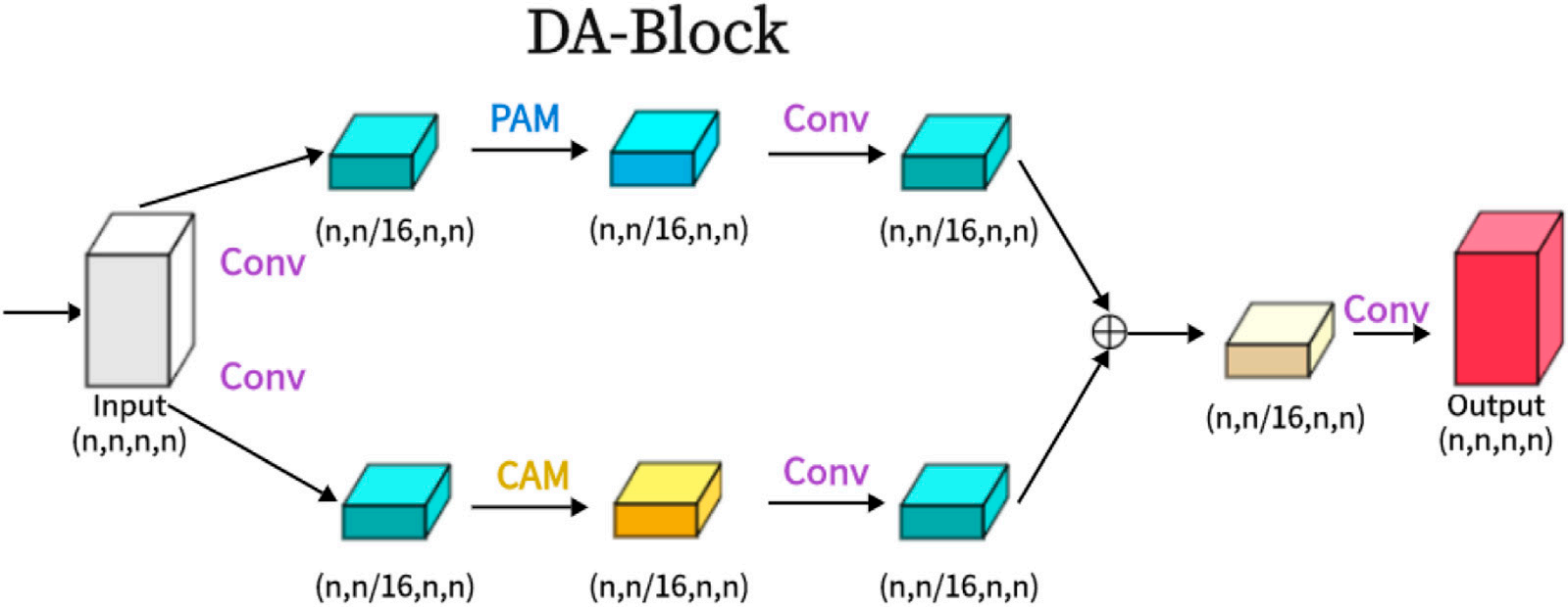
The proposed Dual Attention Block (DA-Block) is shown in the Figure. The same input feature map is input into two feature extraction layers, one is the position feature extraction block and the other is the channel feature extraction block, and finally, the two different features are fused to obtain the final DA-Block output.

As shown in the attached Figure 2, the Dual Attention Block (DA-Block) serves as a feature extraction module that integrates image-specific features of position and channel. This enables feature extraction tailored to the unique attributes of the image. Particularly in the context U-Net shaped architectures, the specialized feature extraction capabilities of the DA-Block are crucial. While Transformers are adept at using attention mechanisms to extract global features, they are not specifically tailored for image-specific attributes. In contrast, the DA-Block excels in both position-based and channel-based feature extraction, enabling a more detailed and accurate set of features to be obtained. Therefore, we incorporate it into the encoder and skip connections to enhance the model’s segmentation performance. The DA-Block consists of two primary components: one featuring a Position Attention Module (PAM), and the other incorporating a Channel Attention Module (CAM), both borrowed from the Dual Attention Network for scene segmentation (Fu et al., 2019).

#### 3.2.1 PAM (position attention module)

**FIGURE 3:**
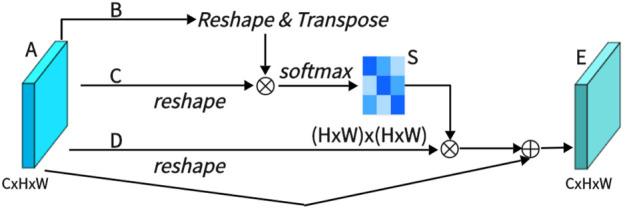
Architecture of position attention Mechanism (PAM).

As shown in Figure 3, PAM captures spatial dependencies between any two positions of feature maps, updating specific features through a weighted sum of all position features. The weights are determined by the feature similarity between two positions. Therefore, PAM is effective at extracting meaningful spatial features.

PAM initially takes a local feature, denoted as A ∈ *R* ^*C*×*H*×*W*^ (C represents Channel, H represents, and W represents Width). We then feed A into a convolutional layer, resulting in three new feature maps, namely, B, C, and D, each of size *R* ^*C*×*H*×*W*^. Next, we reshape B and C to *R* ^*C*×*N*^, where N = H× W denotes the number of pixels. We perform a matrix multiplication between the transpose of C and B and subsequently use a softmax layer to compute the spatial attention map S ∈ *R* ^*N*×*N*^:

$$S_{ji}=\frac{\exp\left(B_{i}\cdot C_{j}\right)}{\sum_{i=1}^{N}\exp\left(B_{i}\cdot C_{j}\right)}$$ (1)

Here, *S*_*ji*_ measures the impact of the i-th position on the j-th position. We then reshape matrix D to *R* ^*C*×*N*^. A matrix multiplication is performed between D and the transpose of S, followed by reshaping the result to *R* ^*C*×*H*×*W*^. Finally, we multiply it by a parameter *α* and perform an element-wise sum operation with the features A to obtain the final output E ∈ *R* ^*C*×*H*×*W*^:

$$E_{j}=\alpha \overset{N}{\underset{i=1}{\sum }}\left(S_{ji}D_{i}\right)+A_{j}$$ (2)

The weight *α* is initialized as 0 and is learned progressively. PAM has a strong capability to extract spatial features. It can be inferred from Eq. 2 that the resulting feature E at each position is a weighted sum of the features across all positions and original features, it possesses global contextual features and aggregates context based on the spatial attention map. This ensures effective extraction of position features while maintaining global contextual information.

#### 3.2.2 CAM (channel attention module)

**FIGURE 4:**
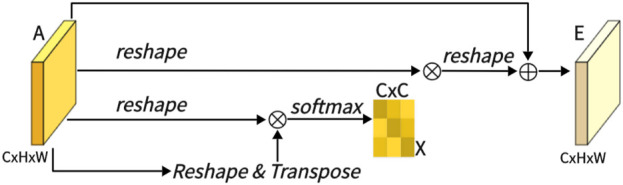
Architecture of channel attention Mechanism (CAM).

As shown in Figure 4, this is CAM, which excels in extracting channel features. Unlike PAM, we directly reshape the original feature A ∈ *R* ^*C*×*H*×*W*^ to *R* ^*C*×*N*^, and then perform a matrix multiplication between A and its transpose. Subsequently, we apply a softmax layer to obtain the channel attention map X ∈ *R* ^*C*×*C*^:

$$X_{ji}=\frac{\exp\left(A_{i}\cdot A_{j}\right)}{\sum_{i=1}^{C}\exp\left(A_{i}\cdot A_{j}\right)}$$ (3)

Here, *x*_*ji*_ measures the impact of the i-th channel on the j-th channel. Next, we perform a matrix multiplication between the transpose of X and A, reshaping the result to *R* ^*C*×*H*×*W*^. We then multiply the result by a scale parameter *β* and perform an element-wise sum operation with A to obtain the final output E ∈ *R* ^*C*×*H*×*W*^:

$$E_{j}=\beta \overset{N}{\underset{i=1}{\sum }}\left(X_{ji}A_{i}\right)+Aj$$ (4)

Like *α*, *β* is learned through training. Similar to PAM, during the extraction of channel features in CAM, the final feature for each channel is generated as a weighted sum of all channels and original features, thus endowing CAM with powerful channel feature extraction capabilities.

#### 3.2.3 DA (dual attention module)

As shown in the Figure 2, we present the architecture of the Dual Attention Block (DA-Block). This architecture merges the robust position feature extraction capabilities of the Position Attention Module (PAM) with the channel feature extraction strengths of the Channel Attention Module (CAM). Furthermore, when coupled with the nuances of traditional convolutional methodologies, the DA-Block emerges with superior feature extraction capabilities. DA-Block consists of two components, the first one is dominated by PAM and the second one is dominated by CAM. The first component takes the input features and performs one convolution to scale the number of channels by one-sixteenth to get *α* ^1^. This convolution operation not only simplifies feature extraction by PAM but also helps to adjust the scale and dimension of features, making them more suitable for the subsequent attention mechanism computations. Following a PAM feature extraction and another convolution, $\hat{\alpha^{1}}$ is obtained, which further refines the extracted features.

$$\alpha^{1}=Conv\left(input\right)$$ (5)

$$\hat{\alpha^{1}}=Conv\left(PAM\left(\alpha^{1}\right)\right)$$ (6)

The other component is the same, with the only difference being that the PAM block is replaced with a CAM with the following formula:

$$\alpha^{2}=Conv\left(input\right)$$ (7)

$$\hat{\alpha^{2}}=Conv\left(CAM\left(\alpha^{2}\right)\right)$$ (8)

After extracting $\hat{\alpha^{1}}$ and $\hat{\alpha^{2}}$ from the two layers of attention, the output is obtained by aggregating and summing the two layers of attention and recovering the number of channels in one convolution.

$$output=Conv\left(\hat{\alpha^{1}}+\hat{\alpha^{2}}\right)$$ (9)

To optimize the DA-Block for medical image segmentation, we fine-tuned the number of intermediate channels. This optimization allows the model to focus on the most critical features, enhancing its sensitivity to key information in the medical images. By adapting the DA-Block to the specific characteristics of medical images, we enable the model to better capture the fine-grained details necessary for accurate segmentation. This targeted optimization sets our approach apart from previous works, which often overlook the importance of tailoring attention mechanisms to the unique demands of medical image segmentation.

### 3.3 Encoder with transformer and dual attention

As illustrated in Figure 1, the encoder architecture consists of four key components: convolution blocks, DA-Block, embedding layers, and transformer layers. Of particular significance is the inclusion of the DA block before the Transformer layer. This design is aimed at performing specialized image processing on the post-convolution features, enhancing the Transformer’s feature extraction for image content. While the Transformer architecture plays a crucial role in preserving global context, the DA block strengthens the Transformer’s capability to capture image-specific features, enhancing its ability to capture global contextual information in the image. This approach effectively combines global features with image-specific spatial and channel characteristics.

The first component comprises the three convolutional blocks of the architecture of the U-Net and its diverse iterations, seamlessly integrating convolutional operations with downsampling processes. Each convolutional layer halves the size of the input feature map and doubles its dimension, a configuration empirically found to maximize feature expressiveness while maintaining computational efficiency. The second component uses DA-Block extract features at both positional and channel levels, enhancing the depth of feature representation while preserving the intrinsic characteristics of the input map. The third component is the embedding layer serves as a critical intermediary, enabling the requisite dimensional adaptation, a prelude to the subsequent Transformer strata. The fourth component integrates Transformer layers for enhanced global feature extraction, beyond the reach of traditional CNNs. Putting the above parts together, it works as follows: the input image traverses three consecutive convolutional blocks, systematically expanding the receptive field to encompass vital features. Subsequently, the DA-Block refines features through the application of both position-based and channel-based attention mechanisms. Following this, the remodeled features undergo a dimensionality transformation courtesy of the embedding stratum before they are channeled into the Transformer framework for the extraction of all-encompassing global features. This orchestrated progression safeguards the comprehensive retention of information across the continuum of successive convolutional layers. Ultimately, the Transformer-generated feature map is restructured and navigated through skip connection layers to feed into the decoder.

By combining convolutional neural networks, transformer architectures, and dual-attention mechanisms, the encoder configuration culminates in a robust capability for feature extraction, resulting in a symbiotic powerhouse of capabilities.

### 3.4 Skip-connections with dual attention

Similar to other U-structured models, we have also incorporated skip connections between the encoder and decoder to bridge the semantic gap that exists between them. To further minimize this semantic gap, we introduced dual-attention blocks (DA-Blocks), as depicted in Figure 1, in each of the three skip connection layers. This decision was based on our observation that traditional skip connections often transmit redundant features, which DA-Blocks effectively filter. Integrating DA-Blocks into the skip connections allows them to refine the sparsely encoded features from both positional and channel perspectives, extracting more valuable information while reducing redundancy. By doing so, DA-Blocks assist the decoder in more accurate feature map reconstruction. Moreover, the inclusion of DA-Blocks not only enhances the model’s robustness but also effectively mitigates sensitivity to overfitting, contributing to the overall performance and generalization capability of the model.

### 3.5 Decoder

As depicted in Figure 1, the right half of the diagram corresponds to the decoder. The primary role of the decoder is to reconstruct the original feature map by utilizing features acquired from the encoder and those received through skip connections, employing operations like upsampling.

The decoder’s components include feature fusion, a segmentation head, and three upsampling convolution blocks. The first component: feature fusion entails the integration of feature maps transmitted through skip connections with the existing feature maps, thereby assisting the decoder in faithfully reconstructing the original feature map. The second component: the segmentation head is responsible for restoring the final output feature map to its original dimensions. The third component: the three upsampling convolution blocks incrementally double the size of the input feature map in each step, effectively restoring the image’s resolution.

Putting the above parts together, the workflow begins by passing the input image through convolution blocks and subsequently performing upsampling to augment the size of the feature maps. These feature maps undergo a twofold size increase while their dimensions are reduced by half. The features received through the skip connections are then fused, followed by continued upsampling and convolution. After three iterations of this process, the generated feature map undergoes one final round of upsampling and is accurately restored to its original size by the segmentation head.

Thanks to this architecture, the decoder demonstrates robust decoding capabilities, effectively revitalizing the original feature map using features from both the encoder and skip connections.

Furthermore, compared to other transformer-based approaches that extensively utilize transformer blocks throughout the architecture, such as Swin-Unet, DA-TransUNet achieves a more favorable balance between performance and computational efficiency. The judicious integration of DA-Blocks in the encoder and skip connections allows DA-TransUNet to enhance feature representation while maintaining a manageable computational footprint.

## 4 Experiments

To evaluate the proposed method, we performed experiments on Synapse (Landman et al., 2015), CVC-ClinicDB dataset (Bernal et al., 2015), Chest X-ray mask and label dataset (Candemir et al., 2013; Jaeger et al., 2013) Analysis, Kvasir SEG dataset (Jha et al., 2020), Kvasir-Instrument dataset (Jha et al., 2021), 2018ISIC-Task (Tschandl et al., 2018; Codella et al., 2019). The experimental results demonstrate that DA-TransUNet outperforms existing methods across all six datasets. In the following subsections, we first introduce the dataset and implementation details. Then show the results on each of the six datasets.

### 4.1 Datasets

#### 4.1.1 Synapse

The Synapse dataset consists of 30 scans of eight abdominal organs. These eight organs include the left kidney, right kidney, aorta, spleen, gallbladder, liver, stomach and pancreas. There are a total of 3779 axially enhanced abdominal clinical CT images.

#### 4.1.2 CVC—ClinicDB

CVC-ClinicDB is a database of frames extracted from colonoscopy videos, which is part of the Endoscopic Vision Challenge. This is a dataset of endoscopic colonoscopy frames for the detection of polyps. CVC-ClinicDB contains 612 still images from 29 different sequences. Each image has its associated manually annotated ground truth covering the polyp.

#### 4.1.3 Chest Xray

Chest Xray Masks and Labels X-ray images and corresponding masks are provided. The X-rays were obtained from the Montgomery County Department of Health and Human Services Tuberculosis Control Program, Montgomery County, Maryland, United States. The set of images contains 80 anterior and posterior X-rays, of which 58 X-rays are normal and 1702 X-rays are abnormal with evidence of tuberculosis. All images have been de-identified and presented in DICOM format. The set contains a variety of abnormalities, including exudates and corneal morphology. It contains 138 posterior-anterior radiographs, of which 80 radiographs were normal and 58 radiographs showed abnormal manifestations of tuberculosis.

#### 4.1.4 Kvasir SEG

Kvasir SEG is an open-access dataset of gastrointestinal polyp images and corresponding segmentation masks, manually annotated and verified by an experienced gastroenterologist. It contains 1000 polyp images and their corresponding groudtruth, the resolution of the images contained in Kvasir-SEG varies from 332 × 487 to 1920 × 1072 pixels, and the file format is jpg.

#### 4.1.5 Kvasir-instrument

Kvasir-Instrument a gastrointestinal instrument Dataset. It contains 590 endoscopic tool images and their groud truth mask, the resolution of the image in the dataset varies from 720 × 576 to 1280 × 1024, which consists of 590 annotated frames comprising of GI procedure tools such as snares, balloons, biopsy forceps, etc. The file format is jpg.

#### 4.1.6 2018ISIC-task

The dataset used in the 2018 ISIC Challenge addresses the challenges of skin diseases. It comprises a total of 2512 images, with a file format of JPG. The images of lesions were obtained using various dermatoscopic techniques from different anatomical sites (excluding mucous membranes and nails). These images are sourced from historical samples of patients undergoing skin cancer screening at multiple institutions. Each lesion image contains only a primary lesion.

### 4.2 Implementation settings

#### 4.2.1 Baselines

In our endeavor to innovate in the field of medical image segmentation, we benchmark our proposed model against an array of highly-regarded baselines, including the U-net, UNet++, DA-Unet, Attention U-net, and TransUNet. The U-net has been a foundational model in biomedical image segmentation (Ronneberger et al., 2015). Unet++ brings added sophistication with its implementation of intermediate layers (Zhou et al., 2018). The DA-Unet goes a step further by integrating dual attention blocks, amplifying the richness of features extracted (Cai et al., 2022). The Attention U-net employs an attention mechanism for improved feature map weighting (Oktay et al., 2018), and finally, the TransUNet deploys a transformer architecture, setting a new bar in segmentation precision (Chen et al., 2021). Through this comprehensive comparison with these eminent baselines, we aim to highlight the unique strengths and expansive potential applications of our proposed model. Additionally, we benchmarked our model against advanced state-of-the-art algorithms. UCTansNet allocates skip connections through the attention module in the traditional U-net model (Wang et al., 2022a). TransNorm integrates the Transformer module into the encoder and skip connections of standard U-Net (Azad et al., 2022b). A novel Transformer module was designed and a model named MIM was built with it (Wang et al., 2022b). By extensively comparing our model with current state-of-the-art solutions, we intend to showcase its superior segmentation performance.

#### 4.2.2 Implementation details

We implemented DA-TransUNet using the PyTorch framework and trained it on a single NVIDIA RTX 3090 GPU (Paszke et al., 2019). The model was trained with an image resolution of 256 × 256 and a patch size of 16. We employed the Adam optimizer, configured with a learning rate of 1e-3, momentum of 0.9, and weight decay of 1e-4. All models were trained for 500 epochs unless stated otherwise. In order to ensure the convergence of the indicators, but due to different data set sizes, we used 50 epochs for training on the two data sets, Chest Xray Masks and Labels and ISIC 2018-Task.

During the training phase on five datasets, including CVC-ClinicDB, the proposed DA-TransUNet model is trained in an end-to-end manner. Its objective function consists of a weighted binary cross-entropy loss function (BCE) and a Dice coefficient loss function. To facilitate training, the final loss function, termed “Loss,” is formulated as follows:

$$\text{Loss}=\frac{1}{2}\times \text{BCE}+\frac{1}{2}\times \text{DiceLoss}$$ (10)

To ensure a fair evaluation of the Synapse dataset, we utilized the pre-trained model “R50-ViT” with input resolution and patch size set to 224 × 224 and 16, respectively. We trained the model using the SGD optimizer, setting the learning rate to 0.01, momentum of 0.9, and weight decay of 1e-4. The default batch size was set to 24. The loss function employed for the Synapse dataset is defined as follows:

$$\text{Loss}=\frac{1}{2}\times \text{Cross}-\text{Entropy Loss}+\frac{1}{2}\times \text{DiceLoss}$$ (11)

This loss function balances the contributions of cross-entropy and Dice losses, ensuring impartial evaluation during testing on the Synapse dataset.

When using the datasets, we use a 3 to 1 ratio, where 75% is the training set and 25% is the test set, to ensure adequacy of training.

#### 4.2.3 Model evaluation

In evaluating the performance of DA-TransUNet, we utilize a comprehensive set of metrics including Intersection over Union (IoU), Dice Coefficient (DSC), and Hausdorff Distance (HD). These metrics are industry standards in computer vision and medical image segmentation, providing a multifaceted assessment of the model’s accuracy, precision, and robustness.

The choice of these metrics is based on their complementary nature and ability to capture different aspects of segmentation quality. IoU and DSC measure the overlap between the predicted and ground truth segmentation masks, providing a global assessment of the model’s ability to accurately identify and delineate target structures. HD, on the other hand, captures the maximum distance between the predicted and ground truth segmentation boundaries, ensuring that the predicted segmentation closely adheres to the true boundaries of the target structures, even in the presence of small segmentation errors or irregularities.

IOU (Intersection over Union) is one of the commonly used metrics to evaluate the performance of computer vision tasks such as object detection, image segmentation and instance segmentation. It measures the degree of overlap between the predicted region of the model and the actual target region, which helps us to understand the accuracy and precision of the model. In target detection tasks, IOU is usually used to determine the degree of overlap between the predicted bounding box (Bounding Box) and the real bounding box. In image segmentation and instance segmentation tasks, IOU is used to evaluate the degree of overlap between the predicted region and the ground truth segmentation region.

$$IOU=\frac{TP}{FP+TP+FN}$$ (12)

The Dice coefficient (also known as the Sørensen-Dice coefficient, F1-score, DSC) is a measure of model performance in image segmentation tasks, and is particularly useful for dealing with class imbalance problems. It measures the degree of overlap between the predicted results and the ground truth segmentation results, and is particularly effective when dealing with segmentation of objects with unclear boundaries. The Dice coefficient is commonly used as a measure of the model’s accuracy on the target region in image segmentation tasks, and is particularly suitable for dealing with relatively small or uneven target regions.

$$Dice\left(P,T\right)=\frac{\left|P_{1}\cap T_{1}\right|}{\left|P_{1}|+|T_{1}\right|}\Leftrightarrow \text{Dice}=\frac{2|T\cap P|}{\left|F|+|P\right|}$$ (13)

Hausdorff Distance (HD) is a distance measure for measuring the similarity between two sets and is commonly used to evaluate the performance of models in image segmentation tasks. It is particularly useful in the field of medical image segmentation to quantify the difference between predicted and true segmentations. The computation of Hausdorff distance captures the maximum difference between the true segmentation result and the predicted segmentation result, and is particularly suitable for evaluating the performance of segmentation models in boundary regions.

$$H\left(A,B\right)=\max\left\{\max_{a\in A}\min_{b\in B}\|a-b\|,\max_{b\in B}\min_{a\in A}\|b-a\|\right\}$$ (14)

We evaluate using both Dice and HD in the Synapse dataset and both Dice and IOU in other datasets.

### 4.3 Comparison to the state-of-the-art methods

#### 4.3.1 Segmentation performance and comparison

We have chosen U-net (Ronneberger et al., 2015), Res-Unet (Diakogiannis et al., 2020), TransUNet (Chen et al., 2021), U-Net++(Zhou et al., 2018), Att-Unet (Oktay et al., 2018), TransNorm (Azad et al., 2022b), UCTransNet (Wang et al., 2022a), MultiResUNet (Ibtehaz and Rahman, 2020), swin-unet (Cao et al., 2022) and MIM (Wang et al., 2022b) to compare with our DA-TransUNet, and the experimental data are tabulated below.

In order to demonstrate the superiority of the DA-TransUNet model proposed in this paper, we conducted the main experiments using the Synapse dataset and compared it with its 11 state-of-the-art models (SOTA) (see Table1).

**TABLE 1:** Experimental results on the Synapse dataset.

Model	Year	DSC *↑ (%)*	HD *↓*	Aorta	Gallbladder	Kidney(L)	Kidney(R)	Liver	Pancreas	Spleen	Stomach
U-net (Ronneberger et al., 2015)	2015	76.85	39.70	89.07	**69.72**	77.77	68.6	93.43	53.98	86.67	75.58
U-Net++(Zhou et al., 2018)	2018	76.91	36.93	88.19	68.89	81.76	75.27	93.01	58.20	83.44	70.52
Residual U-Net (Diakogiannis et al., 2020)	2018	76.95	38.44	87.06	66.05	**83.43**	76.83	93.99	51.86	85.25	70.13
Att-Unet (Oktay et al., 2018)	2018	77.77	36.02	**89.55**	68.88	77.98	71.11	93.57	58.04	87.30	75.75
MultiResUNet (Ibtehaz and Rahman, 2020)	2020	77.42	36.84	87.73	65.67	82.08	70.43	93.49	60.09	85.23	75.66
TransUNet (Chen et al., 2021)	2021	77.48	31.69	87.23	63.13	81.87	77.02	94.08	55.86	85.08	75.62
UCTransNet (Wang et al., 2022a)	2022	78.23	26.75	84.25	64.65	82.35	77.65	94.36	58.18	84.74	79.66
TransNorm (Azad et al., 2022b)	2022	78.40	30.25	86.23	65.1	82.18	78.63	94.22	55.34	89.50	76.01
MIM(Wang et al., 2022b)	2022	78.59	26.59	87.92	64.99	81.47	77.29	93.06	59.46	87.75	76.81
swin-unet (Cao et al., 2022)	2022	79.13	**21.55**	85.47	66.53	83.28	79.61	94.29	56.58	**90.66**	76.60
**DA-TransUNet(Ours)**	2023	**79.80**	23.48	86.54	65.27	81.70	**80.45**	**94.57**	**61.62**	88.53	**79.73**
**Average Relative Improvement**	-	**2.03**	**−9.00**	**−0.73**%	**−1.09**%	**0.28**%	**5.21**%	**0.82**%	**4.86**%	**1.97**%	**4.5**%

The bold values indicate the best performance among all the methods compared in each respective evaluation metric. Specifically, for each row in a table, the bold number represents the method that achieves the highest score or lowest error on that particular metric, demonstrating its superior performance relative to the other approaches.

**FIGURE 5:**
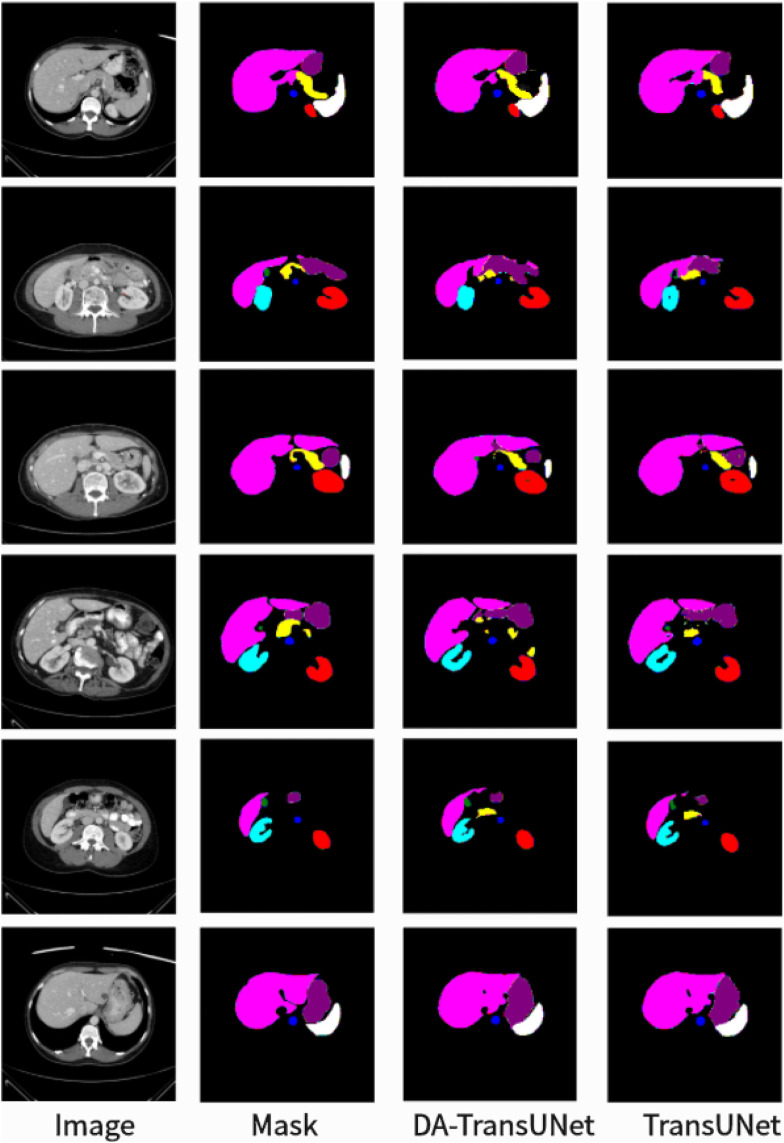
Segmentation results of TransUNet and DA-TransUNet on the Synapse dataset.

**FIGURE 6:**
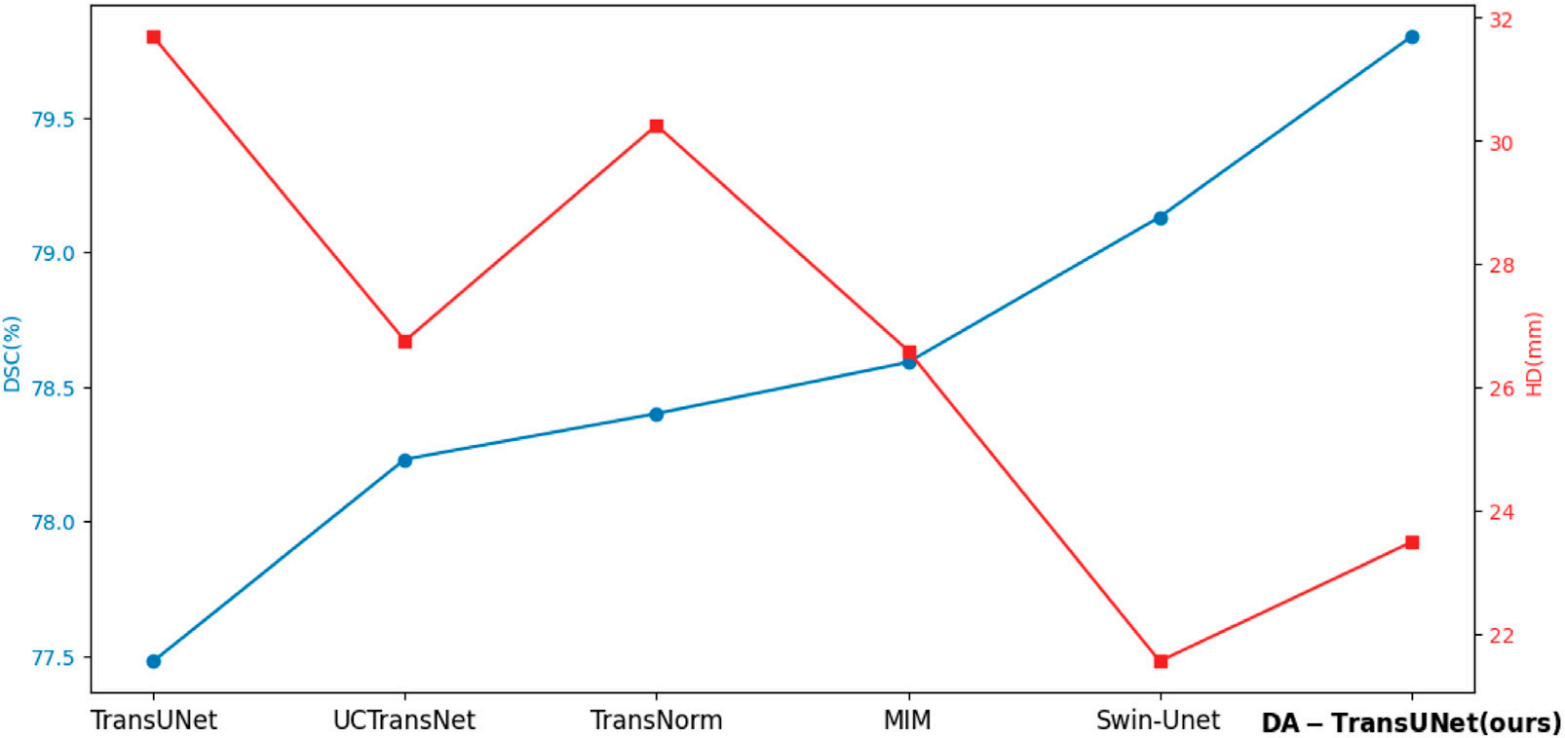
Line chart of DSC and HD values of several advanced models in the Synapse dataset.

As shown in the Figure 5, we can see that the average DSC and average HD evaluation criteria are 79.80% and 23.48 mm, respectively, which are improved by 2.32% and 8.21 mm, respectively, compared with TransUNet, which indicates that our DA-TransUNet has better segmentation ability than TransUNer in terms of overall segmentation results and organ edge prediction. As shown in the Figure 6, on the other hand, we can see that DSC has the highest value of our model. Although HD is higher than Swin-Unet, it is still an improvement compared to several newer models and TransUNet. The segmentation time for an image is 35.98 ms for our DA-TransUNet and 33.58 ms for TransUNet, which indicates that there is not much difference in the segmentation speed between the two models, but our DA-TransUNet has better segmentation results. In the segmentation results of 8 organs, DA-TransUNet outperforms TransUNet by 2.14%, 3.43%, 0.48%, 3.45%, and 4.11% for the five datasets of Gallbladder, right kidney, liver, spleen, and stomach, respectively. The segmentation rate for the pancreas is notably higher at 5.73%. In a comparative evaluation across six distinct organs, DA-TransUNet demonstrates superior segmentation capabilities relative to TransUNet. Nevertheless, it exhibits a marginal decrement in the segmentation accuracy for the aorta and left kidney by 0.69% and 0.17%, respectively. The model achieves the best segmentation rates for the right kidney, liver, pancreas, and stomach, indicating superior feature learning capabilities on these organs.

To further confirm the better segmentation of our model compared to TransUNet, we visualized the segmentation plots of TransUNet and DA-TransUNet (see Figure 5). From the yellow and purple parts in the first column, we can see that our segmentation effect is obviously better than that of TransUNet; from the second column, the extension of purple is better than that of TransUNet, and there is no vacancy in the blue part; from the third column, there is a semicircle in the yellow part, and the vacancy in red is smaller than that of TransUNet, etc. It is evident that DA-TransUNet outperforms TransUNet in segmentation quality. In summary, DA-TransUNet significantly surpasses TransUNet in segmenting the left kidney, right kidney, spleen, stomach, and pancreas. It also offers superior visualization performance in image segmentation.

We simultaneously took DA-TransUNet in five datasets, CVC-ClinicDB, Chest Xray Masks and Labels, ISIC2018-Task, kvasir-instrument, and kvasir-seg, and compared it with some classical models (see Table 2). In the table, the values of IOU and Dice of DA-TransUNet are higher than TransUNet in all five datasets, CVC-ClinicDB, Chest Xray Masks and Labels, ISIC2018-Task, kvasir-instrument, and kvasir-seg. Also DA-TransUNet has the best dataset segmentation in four of the five datasets. As seen in the table, our DA-TransUNet has more excellent feature learning and image segmentation capabilities.

**TABLE 2:** Experimental results of datasets (CVC-ClinicDB, Chest Xray Masks and Labels, ISIC2018-Task, kvasir-instrument, kvasir-seg).

	CVC-ClinicDB		Chest xray masks and labels		ISIC2018-task		Kvasir-instrument		Kvasir-seg	
	Iou *↑*	Dice *↑*	Iou *↑*	Dice *↑*	Iou *↑*	Dice *↑*	Iou *↑*	Dice *↑*	Iou *↑*	Dice *↑*
U-net (Ronneberger et al., 2015)	0.7821	0.8693	0.9303	0.9511	0.8114	0.8722	0.8957	0.9358	0.8012	0.8822
Attn-Unet (Oktay et al., 2018)	0.7935	0.8741	0.9274	0.9503	0.8151	0.876	0.8949	0.9359	0.7801	0.8661
Unet++(Zhou et al., 2018)	0.7847	0.8714	0.9289	0.9505	0.8133	0.873	**0.8995**	**0.9389**	0.7767	0.8657
ResUNet (Diakogiannis et al., 2020)	0.5902	0.7422	0.9262	0.9505	0.7651	0.8332	0.8572	0.9141	0.6604	0.7785
TransUNet (Chen et al., 2021)	0.8163	0.8901	0.9301	0.9535	0.8263	0.8878	0.8926	0.9363	0.8003	0.8791
**DA-TransUNet(Ours)**	**0.8251**	**0.8947**	**0.9317**	**0.9538**	**0.8278**	**0.8888**	0.8973	0.9381	**0.8102**	**0.8847**

The bold values indicate the best performance among all the methods compared in each respective evaluation metric. Specifically, for each row in a table, the bold number represents the method that achieves the highest score or lowest error on that particular metric, demonstrating its superior performance relative to the other approaches.

**FIGURE 7:**
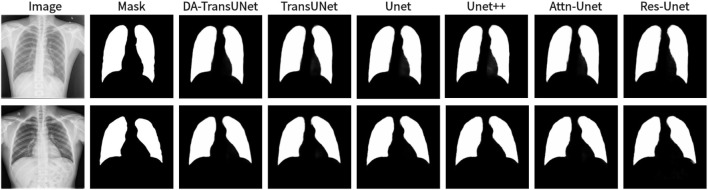
Comparison of qualitative results between DA-TransUNet and existing models on the task of segmenting Chest X-ray Masks and Labels X-ray datasets.

**FIGURE 8:**
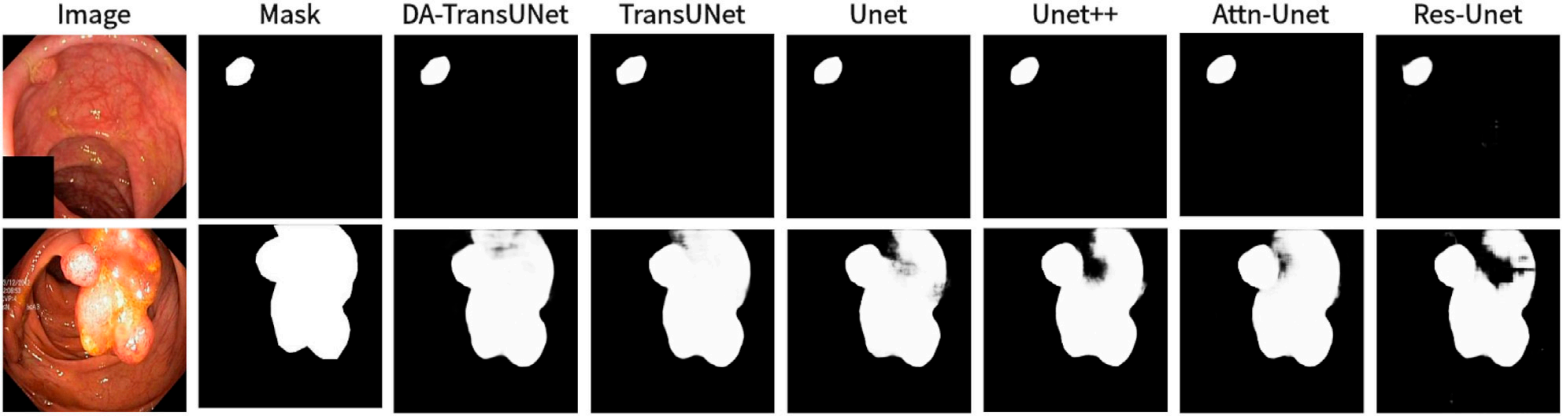
Comparison of qualitative results between DA-TransUNet and existing models on the task of segmenting Kvasir-Seg datasets.

**FIGURE 9:**
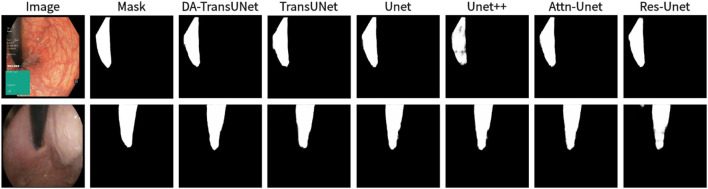
Comparison of qualitative results between DA-TransUNet and existing models on the task of segmenting Kavsir-Instrument datasets.

**FIGURE 10:**
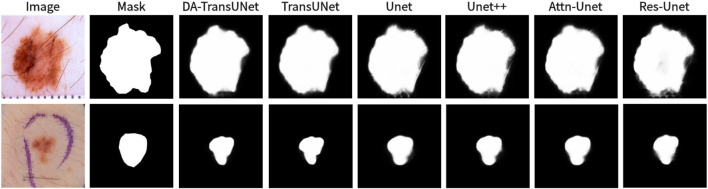
Comparison of qualitative results between DA-TransUNet and existing models on the task of segmenting 2018ISIC-Task datasets.

**FIGURE 11:**
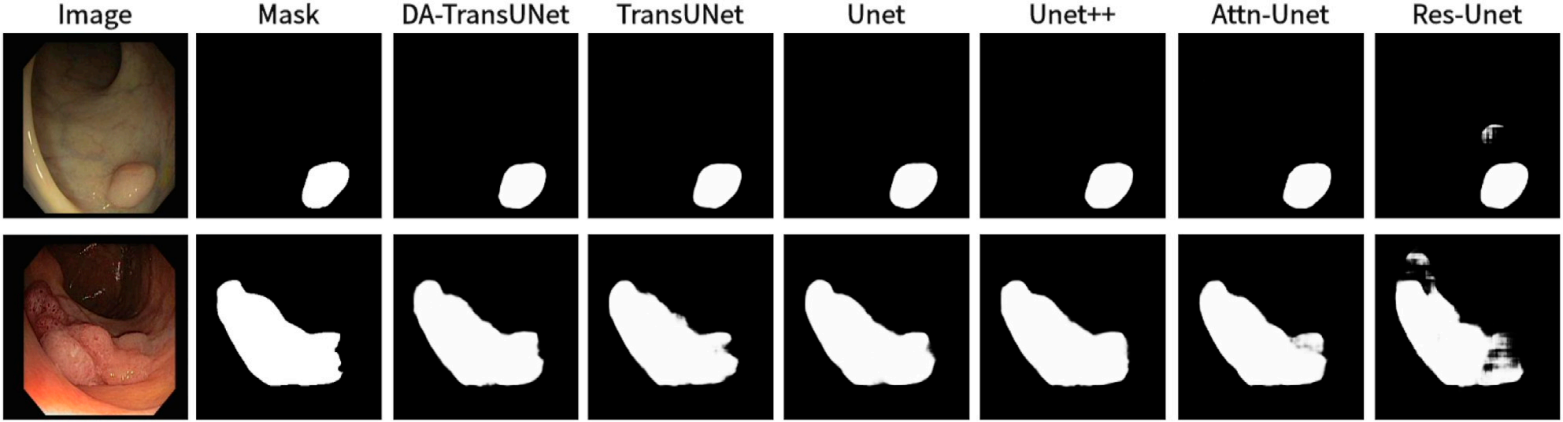
Comparison of qualitative results between DA-TransUNet and existing models on the task of segmenting CVC-ClinicDB datasets.

We also show the results of image segmentation visualization of DA-TransUNet in these five datasets, and we also show the results of the comparison models for the comparison. The visualization results for Chest X-ray Masks and Labels, Kvasir-Seg, Kvasir-Instrument, ISIC2018-Task, and CVC-ClinicDB datasets are presented in Figure 7, Figure 8, Figure 9, Figure 10, and Figure 11, respectively. In the Figure, it can be seen that the segmentation effect of DA-TransUNet has a good performance. Firstly, DA-TransUNet has better segmentation results than TransUNet. In addition, compared with the four classical models of U-net, Unet++, Attn-Unet, and Res-Unet, DA-TransUNet has a certain improvement. It can be seen that the effectiveness of DA-TransUNet for model segmentation is not only confirmed in the Synapse dataset, but also in the five datasets (CVC-ClinicDB, Chest Xray Masks and Labels, ISIC2018-Task, kvasir-instrument, kvasir-seg). We further establish that DA-TransUNet excels in both 3D and 2D medical image segmentation.

#### 4.3.2 Computational complexity and efficiency

The integration of DA-Blocks in the encoder and skip connections introduces additional computational overhead compared to the standard TransUNet architecture. Let the input feature map have a spatial resolution of *H* × *W* and *C* channels. The computational complexity of the Position Attention Module (PAM) is $O\left(H^{2}W^{2}C\right)$ , while the Channel Attention Module (CAM) has a complexity of $O\left(C^{2}HW\right)$ . As the DA-Block consists of both PAM and CAM, its overall computational complexity is $O\left(H^{2}W^{2}C+C^{2}HW\right)$ . However, it is worth noting that the DA-Block itself is not computationally intensive, as it only involves simple matrix multiplications and element-wise operations.

Table 3 compares the number of parameters, Dice Similarity Coefficient (DSC), and Hausdorff Distance (HD) between DA-TransUNet and TransUNet. The incorporation of DA-Blocks leads to a modest increase of 2.54% in the number of parameters compared to TransUNet. This incremental increase in parameters is justifiable considering the substantial performance gains achieved by DA-TransUNet, as demonstrated in our experimental results (Section 4). DA-TransUNet achieves an average improvement of 2.99% in DSC and 25.9% in HD compared to TransUNet. The strategic placement of DA-Blocks allows for efficient feature refinement while maintaining a reasonable model size.

**TABLE 3:** Comparison of model parameters and performance between DA-TransUNet and TransUNet.

Model	Params	Params increase	DSC improvement	HD improvement
TransUNet	105,276,066	-	-	-
DA-TransUNet	**107,950,840**	**2.54%**	**2.99%**	**25.9%**

The bold values indicate the best performance among all the methods compared in each respective evaluation metric. Specifically, for each row in a table, the bold number represents the method that achieves the highest score or lowest error on that particular metric, demonstrating its superior performance relative to the other approaches.

### 4.4 Ablation study

We conducted ablation experiments on the DA-TransUNet model using the Synapse dataset to discuss the effects of different factors on model performance. Specifically, it includes: 1) DA-Block in Encoder. 2) DA-Block in Skip Connection.

#### 4.4.1 Effect of the DA-Block in encoder and skip connection

In this research (see Table 4), we conducted experiments to assess the impact of integrating DA-Blocks into the encoder and skip connections on the model’s segmentation performance. To be specific, we introduced DA-Blocks into each layer of the skip connections. The results demonstrated an improvement: the DSC baseline saw an increase from 77.48% to 78.28%, HD index dropped from 31.69 mm to 29.09 mm. This indicates that the addition of DA-Blocks at each skip connection layer provided the decoder with more refined features, mitigating feature loss during the upsampling process, thereby reducing the risk of overfitting and enhancing model stability. Furthermore, incorporating DA-Blocks into the encoder before the Transformer yielded an enhancement, with the DSC baseline increasing from 77.48% to 78.87%, even though the HD metric decreased from 31.69 mm to 27.71 mm. In conclusion, based on the findings presented in Table 4, we can assert that the inclusion of DA-Blocks both before the Transformer layer and within the skip connections effectively boosts medical image segmentation capabilities.

**TABLE 4:** Effects of combinatorial placement of DA-Blocks in the encoder and through skip connections on performance metrics.

	Encoder with DA	Skip with DA	DSC *↑*	HD *↓*
DA-TransUNet			77.48	31.69
DA-TransUNet		*√*	78.28	29.09
DA-TransUNet	*√*		78.87	27.71
DA-TransUNet	*√*	*√*	**79.80**	**23.48**

The bold values indicate the best performance among all the methods compared in each respective evaluation metric. Specifically, for each row in a table, the bold number represents the method that achieves the highest score or lowest error on that particular metric, demonstrating its superior performance relative to the other approaches.

#### 4.4.2 Effect of adding DA-Blocks to skip connections in different layers

Building on the quantitative results from Table 5, we experimented with various configurations of DA-Block placement across three different layers of skip connections to identify the optimal architectural layout for enhancing the model’s performance. Specifically, when DA-Blocks were added to just the first layer, the DSC metric improved to 79.36% from a baseline of 78.87%, and the HD metric decreased to 25.80 mm from 27.71 mm. Adding DA-Blocks to the second and third layers resulted in some progress. When DA-Blocks were integrated across all layers, there was an improvement, reflected by a DSC of 79.80% and a HD of 23.48 mm. In contrast to traditional architectures where skip connections indiscriminately pass features from the encoder to the decoder, our approach with DA-Blocks selectively improves feature quality at each layer. The results, as corroborated by Table 5, reveal that introducing DA-Blocks to even a single layer enhances performance, and the greatest gains are observed when applied across all layers. This indicates the effectiveness of integrating DA-Blocks within skip connections for enhancing both feature extraction and medical image segmentation. Therefore, the table clearly supports the idea that layer-wise inclusion of DA-Blocks in skip connections is an effective strategy for enhancing medical image segmentation.

**TABLE 5:** Effects of incorporating DA-Block in the encoder and skip connections at different layers on performance metrics.

	1st layer	2nd layer	3rd layer	DSC *↑*	HD *↓*
DA-TransUNet				78.87	27.71
DA-TransUNet	*√*			79.36	25.80
DA-TransUNet		*√*		78.65	23.43
DA-TransUNet			*√*	79.49	30.71
DA-TransUNet	*√*	*√*	*√*	**79.80**	**23.48**

The bold values indicate the best performance among all the methods compared in each respective evaluation metric. Specifically, for each row in a table, the bold number represents the method that achieves the highest score or lowest error on that particular metric, demonstrating its superior performance relative to the other approaches.

#### 4.4.3 Effect of the number of intermediate channels in DA-Block

Based on the results shown in the Table 6, we conducted a discussion regarding the size of the intermediate layer in the DA-Block, which demonstrates the effectiveness of convolutional layers from an experimental perspective. The original DA-Block had an intermediate layer size that is one-fourth of the input layer size. However, since its intended application is for road scene segmentation and not specifically tailored for medical image segmentation, we deemed that setting the intermediate layer size to one-fourth of the input layer size might not be suitable for the medical image segmentation domain. As seen in the graph, when we set the intermediate layer size to be the same as the input size, the evaluation results show a DSC of 78.55% and HD of 28.22 mm. In the related research DANet (Fu et al., 2019), where the intermediate layer was set to one-fourth of the input layer, the DSC result was 79.71%, and HD was 25.90 mm. However, when we further reduced the size of the intermediate layer to one-sixteenth of the input layer size, we observed an improvement in DSC to 79.80%, and HD decreased further to 23.48 mm. It is evident that setting the intermediate layer to one-sixteenth of the input layer size is more suitable for medical image segmentation tasks. The reduction in the intermediate layer size can help the model mitigate the risk of overfitting, optimize computational resources, and, given the precision requirements of medical image segmentation tasks, enable the model to focus more on selecting the most crucial features, thereby enhancing sensitivity to critical information for the task.

**TABLE 6:** Effect of the number of intermediate channels in DA-Block.

	1	2	4	8	16	32	DSC *↑*	HD *↓*
DA-TransUNet	*√*						78.55	28.22
DA-TransUNet		*√*					79.35	23.77
DA-TransUNet			*√*				79.71	25.90
DA-TransUNet				*√*			79.35	25.66
DA-TransUNet					*√*		**79.80**	**23.48**
DA-TransUNet						*√*	79.71	24.45

The bold values indicate the best performance among all the methods compared in each respective evaluation metric. Specifically, for each row in a table, the bold number represents the method that achieves the highest score or lowest error on that particular metric, demonstrating its superior performance relative to the other approaches.

## 5 Discussion

In this present study, we have discovered promising outcomes from the integration of DA-Blocks with the Transformer and their combination with skip-connections. Encouraging results were consistently achieved across all six experimental datasets.

### 5.1 Statistical validation of the improvements by DA-TransUNet

To enhance the credibility of our results and further validate the superiority of DA-TransUNet, We evaluated the performance of the models discussed in the Experiment Section 4 (U-Net, TransUNet, and DA-TransUNet) on 12 subsets of the Synapse dataset, constituting 40% of the total data, and obtained their Dice Similarity Coefficients (DSC). It is important to note that both DA-TransUNet and TransUNet are based on the U-Net architecture, which serves as the baseline model. Therefore, using U-Net as the benchmark to assess whether the improvements of DA-TransUNet over TransUNet are significant is a valid approach.

We first assessed the normality of the DSC improvement values for both DA-TransUNet and TransUNet relative to U-Net using the Shapiro-Wilk test. The results showed *p*-values of 0.36 and 0.82 for the improvements of DA-TransUNet and TransUNet, respectively. Since both *p*-values are greater than 0.05, we cannot reject the null hypothesis of normality. This indicates that the DSC improvement values for both DA-TransUNet and TransUNet relative to U-Net can be considered approximately normally distributed. We then performed a paired *t*-test to compare the significance of the improvements. As shown in Table 7, the test yielded a t-statistic of 2.45 and a *p*-value of 0.032, demonstrating a significant difference between the improvements achieved by DA-TransUNet and TransUNet.

**TABLE 7:** Statistical analysis of DSC improvements and model performance.

Model	Mean DSC ± SD	95% CI for DSC
DA-TransUNet	79.80 ± 5.01	[74.79, 84.81]
TransUNet	75.84 ± 6.77	[69.06, 82.61]

Comparison of DSC improvements achieved by DA-TransUNet and TransUNet relative to U-net			
Metric	Mean difference	95% CI for difference	t-Test *p*-value
Improvement	3.96	[0.40, 7.53]	0.032

Moreover, to further quantify the superiority of DA-TransUNet over TransUNet, we calculated the 95% confidence interval for the difference in improvements between DA-TransUNet and TransUNet. The results showed that the mean difference was 3.96, with a standard deviation of 5.61, and the confidence interval was [0.40, 7.53]. This means that, at a 95% confidence level, the magnitude of the difference in DSC improvements between DA-TransUNet and TransUNet lies between 0.40 and 7.53.

To provide a comprehensive overview of the models’ performance, we calculated the 95% confidence intervals for their DSC scores. DA-TransUNet achieved a mean DSC of 79.80 ± 5.01, with a confidence interval of [74.79, 84.81], while TransUNet achieved a mean DSC of 75.84 ± 6.77, with a confidence interval of [69.06, 82.61]. These results, summarized in Table 7, suggest that DA-TransUNet not only achieves higher average performance but also exhibits more consistent results compared to TransUNet.

The statistical analysis, confidence intervals, and the quantification of the relative improvement provide strong evidence for the superiority of DA-TransUNet over TransUNet in the task of medical image segmentation. These results highlight the effectiveness of our proposed approach and its potential to advance the field of medical image analysis.

### 5.2 Enhancing feature extraction and segmentation with DA-Blocks

To start with, drawing from empirical results in Table 4, it is demonstrated that the integration of DA-Block within the encoder significantly enhances the feature extraction capabilities as well as its segmentation performance. In the landscape of computer vision, Vision Transformer (ViT) has been lauded for its robust global feature extraction capabilities (Dosovitskiy et al., 2020). However, its falls short in specialized tasks like medical image segmentation, where attention to image-specific features is crucial. To remedy this, in DA-TransUNet we strategically place DA-Blocks ahead of the Transformer module. These DA-Blocks are tailored to first extract and filter image-specific features, such as spatial positioning and channel attributes. Following this initial feature refinement, the processed data is then fed into the Transformer for enhanced global feature extraction. This approach results in significantly improved feature learning and segmentation performance. In summary, the strategic placement of DA-Blocks prior to the Transformer layer constitutes a pioneering approach that significantly elevates both feature extraction efficacy and medical image segmentation precision.

Morever, building on empirical data in Table 5, our integration of DA-Blocks with skip connections significantly improves semantic continuity and the decoder’s ability to reconstruct accurate feature maps. While traditional U-Net architectures (Ronneberger et al., 2015) utilize skip connections to bridge the semantic gap between encoder and decoder, our novel incorporation of Dual Attention Blocks within the skip-connection layers yields promising results. By incorporating DA-Blocks across skip-connection layers, we focus on relevant features and filter out extraneous information, making the image reconstruction process more efficient and accurate. In summary, the strategic inclusion of DA-Blocks in skip connections represents a groundbreaking approach that not only enhances feature extraction but also improves the model’s performance in medical image segmentation.

Lastly, our extensive evaluation across six diverse medical image segmentation datasets demonstrates the effectiveness and generalizability of the DA-TransUNet. The consistent improvements over state-of-the-art methods (Table 1) highlight the impact of our targeted integration of the DA-Block. Moreover, the ablation studies (4.4) provide valuable insights into the individual contributions of the DA-Block in different components of the architecture. These findings not only underscore the novelty of our approach but also shed light on the importance of strategically integrating attention mechanisms for enhanced medical image segmentation. The DA-TransUNet represents a significant step forward in leveraging the power of attention mechanisms and transformers for accurate and robust segmentation across a wide range of medical imaging modalities. Our work paves the way for further exploration of targeted attention mechanisms in medical image analysis and has the potential to impact clinical decision-making and patient care.

### 5.3 Limitations and future directions

Despite the advantages, our model also has some limitations. Firstly, the introduction of the DA-Blocks contributes to an increase in computational complexity. This added cost could potentially be a hindrance in real-time or resource-constrained applications. Although this increase in parameters is relatively modest considering the performance gains achieved, it could still be a concern in resource-constrained scenarios or when dealing with very large-scale datasets. Secondly, the decoder part of our model retains the original U-Net architecture. While this design choice preserves some of the advantages of U-Net, it also means that the decoder has not been specifically optimized for our application. This leaves room for further research and improvements, particularly in the decoder section of the architecture. Thirdly, one potential limitation of our DA-TransUNet architecture is the risk of losing fine-grained details during the tokenization process, which occurs after the convolution and pooling operations in the encoder. This is particularly concerning for medical images with thin and complex structures, where preserving intricate details is crucial for accurate segmentation. Although our proposed integration of the Dual Attention (DA) module before the Transformer in the encoder and within the skip connections helps mitigate this issue to some extent, as evidenced by the improved segmentation performance, we acknowledge that there may still be room for further enhancement in capturing and retaining fine-grained information.

## 6 Conclusion

In this paper, we innovatively proposed a novel approach to image segmentation by integrating DA-Blocks with the Transformer in the architecture of TransUNet. The DA-Blocks, focusing on image-specific position and channel features, were further integrated into the skip connections to enhance the model’s performance. Our experimental results, validated by an extensive ablation study, showed significant improvements in the model’s performance across various datasets, particularly the Synapse dataset.

Our research revealed the potential of image-special features position and channel (DA-Block) in enhancing the feature extraction capability and global information retention of the Transformer. The integration of DA-Block and Transformer substantially improved the model’s performance without creating redundancy. Furthermore, the introduction of DA-Blocks into skip connections not only effectively bridges the semantic gap between the encoder and decoder, but also refines the feature maps, leading to an enhanced image segmentation performance.

Our model also has some limitations. Firstly, the introduction of DA blocks increases computational complexity. This added cost may pose obstacles for real-time or resource-constrained applications. Secondly, the decoder part of our model retains the original U-Net architecture. Lastly, the utilization of image feature positions and channels is only superficial, with deeper exploration possible.

This study has paved the way for the further use of image-special features position and channel (DA-Block) in the field of medical image segmentation. At the same time, it provides the idea of leveraging image characteristics to achieve high-precision medical image segmentation. Future work may focus on optimizing the decoder part of our architecture and exploring methods to reduce the computational complexity introduced by DA blocks without compromising the model’s performance. We believe our approach can inspire future research in the domain of medical image segmentation and beyond.

## Data Availability

Publicly available datasets were analyzed in this study. This data can be found here: B. Landman, Z. Xu, J. E. Igelsias, M. Styner, T. Langerak, and A. Klein, ‘‘Segmentation outside the cranial vault challenge,’’ in MICCAI: Multi Atlas Labeling Beyond Cranial Vault-Workshop Challenge, 2015. J. Bernal, F. J. Sánchez, G. Fernández-Esparrach, D. Gil, C. Rodríguez, and F. Vilariño, ‘‘Wm-dova maps for accurate polyp highlighting in colonoscopy: Validation vs. saliency maps from physicians,’’ Computerized medical imaging and graphics, vol. 43, pp. 99–111, 2015. N. Codella, V. Rotemberg, P. Tschandl, M. E. Celebi, S. Dusza, D. Gutman, B. Helba, A. Kalloo, K. Liopyris, M. Marchetti et al., ‘‘Skin lesion analysis toward melanoma detection 2018: A challenge hosted by the international skin imaging collaboration (isic),’’ arXiv preprint arXiv:1902.03368, 2019. P. Tschandl, C. Rosendahl, and H. Kittler, ‘‘The ham10000 dataset, a large collection of multi-source dermatoscopic images of common pigmented skin lesions,’’ Scientific data, vol. 5, no. 1, pp. 1–9, 2018. D. Jha, P. H. Smedsrud, M. A. Riegler, P. Halvorsen, T. de Lange, D. Johansen, and H. D. Johansen, ‘‘Kvasir-seg: A segmented polyp dataset,’’ in MultiMedia Modeling: 26th International Conference, MMM 2020, Daejeon, South Korea, January 5–8, 2020, Proceedings, Part II 26. Springer, 2020, pp. 451–462. D. Jha, S. Ali, K. Emanuelsen, S. A. Hicks, V. Thambawita, E. GarciaCeja, M. A. Riegler, T. de Lange, P. T. Schmidt, H. D. Johansen et al., ‘‘Kvasir-instrument: Diagnostic and therapeutic tool segmentation dataset in gastrointestinal endoscopy,’’ in MultiMedia Modeling: 27th International Conference, MMM 2021, Prague, Czech Republic, June 22–24, 2021, Proceedings, Part II 27. Springer, 2021, pp. 218–229. S. Jaeger, A. Karargyris, S. Candemir, L. Folio, J. Siegelman, F. Callaghan, Z. Xue, K. Palaniappan, R. K. Singh, S. Antani et al., ‘‘Automatic tuberculosis screening using chest radiographs,’’ IEEE transactions on medical imaging, vol. 33, no. 2, pp. 233–245, 2013 S. Candemir, S. Jaeger, K. Palaniappan, J. P. Musco, R. K. Singh, Z. Xue, A. Karargyris, S. Antani, G. Thoma, and C. J. McDonald, ‘‘Lung segmentation in chest radiographs using anatomical atlases with nonrigid registration,’’ IEEE transactions on medical imaging, vol. 33, no. 2, pp. 577–590, 2013.
